# Autophagy in Cancer Immunotherapy

**DOI:** 10.3390/cells11192996

**Published:** 2022-09-26

**Authors:** Yuhe Lei, Enxin Zhang, Liangliang Bai, Yingjie Li

**Affiliations:** 1 Department of Pharmacy, Shenzhen Hospital of Guangzhou University of Chinese Medicine, Shenzhen 518032, China; 2 Bao′an Authentic TCM Therapy Hospital, Shenzhen 518126, China; 3 School of Pharmaceutical Sciences, Guangzhou University of Chinese Medicine, Guangzhou 510006, China; 4 Center for Chemical Biology and Drug Discovery, Guangzhou Institutes of Biomedicine and Health, Chinese Academy of Sciences, Guangzhou 510530, China

**Keywords:** autophagy, cancer, immunotherapy

## Abstract

Autophagy is a stress-induced process that eliminates damaged organelles and dysfunctional cargos in cytoplasm, including unfolded proteins. Autophagy is involved in constructing the immunosuppressive microenvironment during tumor initiation and progression. It appears to be one of the most common processes involved in cancer immunotherapy, playing bidirectional roles in immunotherapy. Accumulating evidence suggests that inducing or inhibiting autophagy contributes to immunotherapy efficacy. Hence, exploring autophagy targets and their modifiers to control autophagy in the tumor microenvironment is an emerging strategy to facilitate cancer immunotherapy. This review summarizes recent studies on the role of autophagy in cancer immunotherapy, as well as the molecular targets of autophagy that could wake up the immune response in the tumor microenvironment, aiming to shed light on its immense potential as a therapeutic target to improve immunotherapy.

## 1. Introduction

The rise of tumor immunotherapy has brought new hope for humankind to conquer cancer completely. Immune checkpoint blockade (ICB) therapy, with PD-1/PD-L1 blockade therapy as the most typical representative, has become one of the most popular immunotherapies today, and has significantly changed the current cancer treatment landscape [1]. However, many patients do not respond to these therapies for multiple reasons [2]. Therefore, further insight into the molecular mechanisms underlying the resistance of cancer cells to T-cell killing is needed. As an evolutionarily conserved process, autophagy captures and degrades dysfunctional cellular components under the circumstance of various cellular stresses, including hypoxia and nutrient deprivation [3]. Tumor cells always maintain a higher level of basal autophagy than normal cells. Autophagy plays a significant role in tumor cell survival [4]. Accumulating evidence has demonstrated that the autophagic pathway is also involved in the survival and apoptosis of immune cell subpopulations that affect the differentiation, activation, effector function, and translocation to tumors. At the same time, autophagy affects tumor growth by modulating immune responses. In addition, the pro-tumor effects of autophagy can be eliminated by a combination treatment of ICB and autophagy inhibitors [5]. Thus, autophagy is a complex but promising target in cancer immunotherapy. Understanding the mechanisms of autophagy in immunotherapy may prevent tumor recurrence and benefit patients from immunotherapy.

## 2. The Induction and Regulation of Autophagy

In response to cellular starvation or stresses, autophagy is activated to degrade unfolded proteins, damaged organelles, or intracellular pathogens in lysosomes. The digested products are recycled to support metabolic adaptation and maintain energy homeostasis [6]. Macro-autophagy is referred to as autophagy, and its mechanism has been extensively studied. Autophagy is a complex multistep process including induction, vesicle nucleation and elongation, docking and fusion, and degradation and recycling [7]. Each step of autophagy is tightly regulated by conserved autophagy-related genes (*ATG*) or other genes in the endosomal/lysosomal system [8]. The initiation of autophagic vesicle formation is followed by Unc-51-like kinase (ULK) complex activation. The ULK complex, consisting of ULK family kinase, Atg13, and focal adhesion kinase interacting protein 200 kDa (FIP200), is inhibited by the master cell growth regulator, the target of rapamycin (mTOR), and activated by a major sensor of energy stress, AMP-activated protein kinase (AMPK) [9]. At the stage of initiation, the ULK complex localizes to the phagophore and phosphorylates the Beclin 1-VPS34 complex composed of Beclin 1, Atg14, AMBRA1, VPS34, and VPS15 [10]. The VPS34 lipid kinase converts endoplasmic reticulum (ER) and Golgi complex-derived phosphoinositides to phosphatidylinositol-3-phosphate (PI3P) [11]. The PI3P is then bound with WIPI2B to recruit two protein conjugation systems, Atg7-Atg3 and the Atg5-Atg12-Atg6L1 complex [12]. These two complexes function to conjugate lipidated microtubule-associated protein 1A/1B light chain 3 (LC3) family members to phosphatidylethanolamine (PE) [13]. Atg4 is necessary for transforming pro-LC3 into the active cytosolic isoform LC3-I [14]. LC3-I is then conjugated to PE to produce LC3-II, the lipidated form of LC3. The insertion of LC3-II on the surface of the emerging AV is crucial for specific substrate recognition in the selective degradation process [15]. Additionally, an increasing number of autophagy cargo receptors have been found to use LC3 as a docking site to deliver autophagic cargo to the AVs. It is reported that cargo receptors, including SQSTM1 (p62) and neighbor of BRCA1 (NBR1), directly bind to marked autophagic cargo for degradation through NIX and FAM134B [16]. Curvature and sealing of the AV are facilitated by the cooperation of the LC3-PE system and the Atg5-Atg12 system [17]. AV maturation needs Atg9 to recruit the lipid membrane, which is originated from mitochondria, ER, or Golgi, to close the vesicle with trapped cargo [18]. Subsequently, the AVs fuse with lysosomes to create AV-lysosomes where the cargo loaded by the LC3 binding proteins is degraded by lysosomal enzymes. Then, amino acids or other macromolecules are recycled to support cell growth [19]. This step is mediated by Rab GTPases, SNARE, and HOPS complex [20].

## 3. The Role of Autophagy in Cancer Progression

The main function of autophagy is to deal with a range of intracellular and extracellular stresses through recycling metabolites [21]. Multiple stresses induce autophagy in cancer cells, such as metabolic stress, hypoxia, oxidative stress, and immune signals. In response to nutrient deprivation, autophagy is activated to rewire metabolic pathways under the regulation of AMPK and mTORC1 [22]. AMPK is an energy sensor, whereas mTORC1 is a nutrient sensor. AMPK activation or mTORC1 inhibition results in the phosphorylation of targets in the autophagy pre-initiation complex [9]. Moreover, AMPK may inhibit mTORC1 activity by targeting Raptor, a subunit of mTORC1 [23]. Hypoxia was confirmed to be another factor triggering autophagy since the inhibition of oxidative phosphorylation in mitochondria by hypoxia contributes to the activation of AMPK [24]. Additionally, hypoxia suppresses mTOR signaling to increase the stabilization of hypoxia-induced factor 1α (HIF1α) [25]. HIF1α is a transcription factor that upregulates autophagy promoting genes [26]. Furthermore, hypoxia activates activating transcription factor 4 (ATF4) to maintain high levels of autophagic flux by upregulating LC3B and Atg5 [27]. Accumulating reactive oxygen species (ROS) is an important characteristic of oxidative stress in cancer cells, which facilitates DNA damage and tumor progression [28]. The elevated ROS induces autophagy through mTORC1 suppression or nuclear factor-κB (NF-κB)-mediated p62/SQSTM1 upregulation [29,30].

The role of autophagy in cancer progression has been well established recently. It is widely accepted that autophagy is an endogenous defense and nutrient-scavenging mechanism in response to cellular stresses. Recent findings indicated that selective forms of autophagy represent a quality-control mechanism by regulating some specific organelles and proteins [31]. In the dynamics of tumor development, autophagy plays a tumor-suppressive or tumor-promoting role depending on type, stage, and genetic context of the cancers. The dual effects of autophagy in tumor progression are closely correlated to microenvironment stress and the conditions of immune system [32]. In the earliest stages of tumorigenesis, autonomous autophagy is a protective mechanism to limit tumor initiation by eliminating damaged proteins and organelles that are toxic to cells [33]. Hence, mutation or deletion of *ATGs* contributes to tumor initiation. For example, a mouse model with *BECN1* deletion had a higher percentage of spontaneous tumor formation compared with *BECN1* wild-type mouse [34]. However, autophagy is upregulated in established cancers to cope with cellular stresses such as hypoxia and nutrient deprivation, thus supporting cancer growth, survival, and malignancy to mediate cancer development, drug resistance, and metastasis [8]. Moreover, the autophagic level in aggressive tumors is higher than non-aggressive tumors. At the initial stage of metastasis, autophagy may play a suppressive role by preventing tumor necrosis and restricting inflammatory cell infiltration [35]. However, autophagy may act as a promoter during cancer metastasis by accelerating dissemination of cancer cells and enhancing their colonization in the destination organs [36,37]. The dual effects of autophagy in suppression or promotion of cancer remains controversial and has not been fully elucidated [38].

## 4. Autophagy in Tumor Immune System

Autophagy in cancer cells often occurs in response to cytokines and damage-associated molecular patterns (DAMPs) derived from the tumor microenvironment (TME). Several pattern recognition receptors have been verified as autophagy inducers when they receive extracellular DAMPs, including ATP, DNA complexes, and high-mobility group box 1 protein (HMGB1). [39]. For example, polyinosinic:polycytidylic acid and lipopolysaccharide (LPS) activate Toll-like receptor 3 (TLR3) and TLR4, thus triggering autophagy in non-small cell lung cancer (NSCLC) cells [40]. Another study demonstrated that advanced glycosylation end product-specific receptor (AGER) is crucial for HMGB1-induced autophagy in pancreatic and colon cancer cells [41]. As for cytokines, they participate in autophagy initiation in a context-dependent way. Accumulated evidence suggests that transforming growth factor-β (TGF-β) and interferons (type I and II) induce autophagy in various kinds of cancer cells [42]. For instance, TGF-β activates autophagy in human hepatocellular carcinoma cells by upregulating the levels of Beclin 1, Atg5 and Atg7 [43]. In addition, tumor necrosis factor (TNF) and interleukin-6 (IL-6) were found to be autophagy inducers that are associated with carcinogenesis and tumor progression [44]. As a major mechanism of autophagy-associated tumor progression, the immunosuppressive microenvironment shaped by autophagy is our focus. The immune system is essential for preventing the development and metastasis of tumors and for shaping the tumor response to treatment. The immune system recognizes and eliminates tumor cells by immune surveillance. However, some tumor cells survive because of immune evasion, a process where the immunogenicity of tumor cells is reduced, and the immunosuppressive networks are formed [45]. In TME, changes in the autophagy pathway are observed in cancer cells and immune cells, which shape tumor immunity and affect immunotherapy [17].

**Figure 1:**
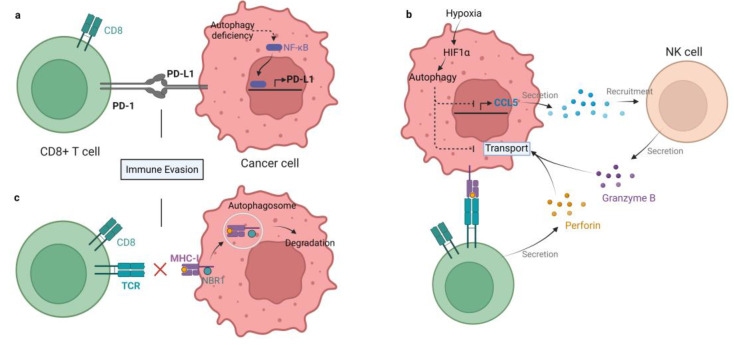
Mechanisms of autophagy-mediated immune evasion. (**a**) Autophagy deficiency in gastric cancer enhances PD-L1 expression by activating NF-κB, therefore promoting PD1/PD-L1-induced immune evasion. (**b**) Hypoxia-induced autophagy impairs killing of breast cancer by degrading NK and CD8+ T cell-derived granzyme B in autophagosomes. In addition, autophagy inhibits chemokine CCL5 production. As a result, the recruitment of NK cells to the TME is blocked. (**c**) MHC-I is recognized by NBR1 and degraded in lysosomes by autophagy in pancreatic cancer cells, which impairs antigen presentation and tumor killing by T cells.

The role of autophagy in immune system activation and immunosuppressive TME formation is complex. On the one hand, autophagy may participate in the process of antigen presentation by APCs through proteasome degradation and the vacuolar pathway [46]. Autophagic degradation can promote APC efficiency by accelerating antigen processing [47]. Autophagy deficiency in pancreatic ductal adenocarcinoma stimulates PD-L1 expression, which may aid in the formation of immunosuppressive TME [48]. Similarly, autophagy inhibition can upregulate PD-L1 expression by accumulating p62/SQSTM1 and activating NF-κB in gastric cancer [49] (Figure 1a). Autophagy contributes to immune evasion by suppressing the innate and adaptive immune response. The inducers of the innate immune response, such as damaged proteins, organelles, and bacteria, are cleared by autophagy. DAMPs and pathogen-associated molecular patterns (PAMPs) are both inducers of innate immunity, which can be captured and degraded in autolysosomes [50]. These facts indicate that autophagy seems to be a key negative regulator of innate immune responses when activated [51]. As a consequence, inhibition of autophagy facilitates the production and secretion of proinflammatory cytokines such as IFNs (type I, II) and TNF-α in vitro and in vivo [52]. Inhibition of autophagy can also trigger programmed cell death of cancer cells, producing DAMPs to activate the adaptive immune system [53]. Moreover, autophagy can suppress the adaptive immune response by weakening T cells’ ability to kill tumor cells [54]. It has been shown that antigenic tumors are recognized and eliminated by T cells when autophagy is inhibited in mice [55]. There are several mechanisms by which autophagy restrains the antitumor T cell response. Autophagy suppresses the functions of NK cells, CD4+ T cells, and CD8+ T cells to help tumor cells combat immunosurveillance, acting as a protective mechanism in tumors [56]. For example, hypoxia-induced autophagy can impair NK cell-mediated killing of breast cancer cells by degrading NK-derived granzyme B in autophagosomes [57] (Figure 1b). Hypoxia-induced autophagy is also reported to attenuate cancer cell sensitivity to CTL by regulating the STAT3 pathway in IGR-Heu lung carcinoma cells [58]. Hence, autophagy in tumors may be a potential target to block immune evasion in the TME. Meanwhile, the initiation of immune response often needs a major histocompatibility complex (MHC) to present antigens to T cells. MHC includes class I and II, that are recognized by CD4+ and CD8+ T cells, respectively [59]. MHC-I presents peptides that prime and activate CD8+ CTLs. Then, CTLs clear the targeted tumor cells by detecting a matching antigen by the MHC-I [60]. The expression of MHC-I is downregulated in pancreatic cancer, resulting in defective antigen presentation, which limits the ability of tumor killing by T cells and immunotherapy. Mechanistically, MHC-I is recognized by NBR1 and degraded in lysosomes through autophagy [55] (Figure 1c). Additionally, in dendritic cells (DCs), autophagy has been shown to accelerate the internalization and degradation of MHC-I. DCs with autophagy inhibition enhance the presentation of viral antigens to CD8+ T cells [61,62]. Recently, hepatic autophagy immune tolerance (HAIT) has been studies and is regarded as another immune evasion mechanism. A study by Laura et al. revealed that autophagy in the liver induces tumor immune tolerance by promoting Treg function and inhibiting T-cell response and interferon-γ production, resulting in the growth of tumors with high tumor mutational burden (TMB). Therefore, autophagy may be a potential target to overcome HAIT [52].

### 4.1. Autophagy in Immune Cells

Tumor development and therapeutic response are influenced by tumor-infiltrating immune cells in TME [63]. A growing number of studies have revealed that autophagy is involved in the differentiation, homeostasis, and development of immune cells [64]. Hence, the alterations of autophagy pathway in immune cells may shape their phenotypes and functions in the TME [65].

#### 4.1.1. Autophagy in T cells

**Figure 2:**
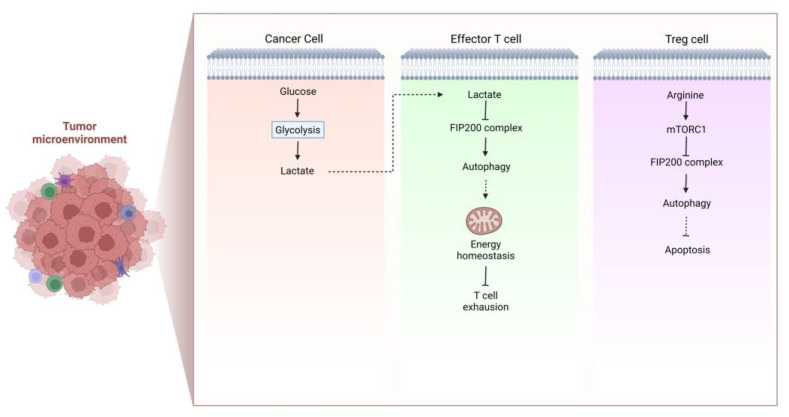
The formation of immunosuppressive TME. Cancer cell-derived lactate lowers FIP200 expression and attenuates autophagy, which is pivotal for energy homeostasis of effector T cells, thus facilitating T cell exhaustion and formation of immunosuppressive TME. In Treg cells, decreased intracellular arginine suppresses mTOR, leading to autophagy activation that is responsible for Treg cell survival. As a consequence, the function of Treg cell is improved to form the immunosuppressive TME.

Autophagy plays a vital role in the survival and proliferation of T cells. In TME of tumor-bearing mice and cancer patients, T cells undergo apoptosis. Xia et al. found that tumor-infiltrating T cells exhibit defective autophagy and a decreased level of FIP200, which is necessary for autophagosome formation. This process is mediated by tumor-derived lactate, which lowers FIP200 expression and causes T cell death by disrupting the balance between pro- and anti-apoptotic factors in the Bcl-2 family to boost immune evasion of cancer [66] (Figure 2). The interactions of T cell receptor (TCR) with stromal cells and IL-7 signaling are essential for naive T cells to survive in the periphery, and it appears that Atg3-dependent autophagy is intrinsically required for these processes [67]. Atg7-deficient T cells have poor proliferative capacity and cannot enter the S phase when TCR is activated. Mechanistically, this is attributed to the accumulation of CDKN1B, which cannot be degraded in autophagy-deficient naive T cells, and negatively regulates cell cycle [68]. Defective autophagy can also disturb T cell activation, differentiation, and stemness. It has been reported that the differentiation from T cells to invariant natural killer T (iNKT) and Treg is facilitated by autophagy [69]. Yang et al. found that deletion of autophagy-related protein PIK3C3/VPS34 reduced the activity of mitochondria upon T cell activation, so that CD4+ T cells failed to differentiate into T helper 1 cells [70]. Similarly, the quality of mitochondria declines when the mitochondrial components cannot be degraded adequately, resulting in ROS accumulation and T cell damage [71]. Depolarized mitochondria were also detected in IL-15-induced resident memory CD8+ T cells with autophagy inhibited by MRT68921 dihydrochloride and 3-methyladenine (3MA), causing T cells exhaustion [72]. CD4+ Treg cells in TME confer another primary mechanism of immune evasion and immunotherapy resistance. Autophagy was shown to participate in maintaining Treg cell function [73]. Inhibition of autophagy by Atg5, Atg7 and AMBRA1 deletion induces Treg cell apoptosis and dysfunction in vivo [74,75,76] (Figure 2).

Autophagy also serves a crucial function in other types of immune cells that interact with T cells. Lysosomal proteolysis of autophagy is necessary for the antigen presentation in dendritic cells [77]. Deleting PIK3C3 (a key player in autophagy) in dendritic cells contributes to reduced CD8α+ dendritic cells and B16 melanoma-specific CTL activity [62]. Myeloid-derived suppressor cells (MDSCs) dampen antitumor immune responses and treatment effectiveness by directly suppressing T cell activation in TME [78]. An elevated level of autophagy has been detected in MDSCs [79]. Blocking autophagy in MDSCs leads to increased MHC-II expression and impaired tumor growth in vivo [80].

#### 4.1.2. Autophagy in B cells

The survival and development of B cells depends on autophagy. For instance, the development of B cells depends on Atg5 [81]. After ligand LPS activation, basal levels of autophagy are required to preserve the survival of a normal number of peripheral B cells [82]. Intrinsic autophagy in B cells is necessary to sustain the normal function of alloreactive B memory cells. Atg7 deletion in B cell blocks B cell autophagy, inhibiting secondary alloantibody responses and decreasing the frequencies of alloreactive B memory cells [83].

#### 4.1.3. Autophagy in Natural Killer (NK) Cells

The survival of iNKT cells requires intrinsic autophagy. Facing viral infection, autophagy is activated with the aid of phosphorylated FoxO1 and Atg7, which promotes the development and function of NKT cells against viral infection [84]. Autophagy-deficient iNKT cells undergo apoptosis in the mitochondrial pathway. Absence of autophagy not only hinders the maturation of iNKT cells by reducing NKT cell proliferation, but also limits the transition of iNKT cells to a quiescent state [85]. Moreover, the secretion of iNKT can be affected by autophagy. Autophagy-deficient iNKT cells release low levels of IL-4 and IFN-γ compared with normal iNKT cells [86].

#### 4.1.4. Autophagy in Dendritic Cells (DCs)

Autophagy is indispensable for cytokine secretion and antigen presentation in dendritic cells (DCs). It has been reported that Atg5 is essential for DCs to activate CD4+ T cells through antigen presentation [84,85]. Atg5 deficiency in DCs reduces the secretion of IL-2 and IFN-γ by CD4+ T cells, but the levels of IL-12, IL-6, and TNF-α are not affected [87]. Moreover, antigen presentation was hampered by Atg5 deficiency through the MHC-II pathway, which may be resulted from the delay of lysosome-phagosome fusion [84,85,86,87,88,89].

#### 4.1.5. Autophagy in Macrophages

The generation of macrophages requires autophagy at different stages. It is well accepted that tumor-associated macrophages (TAMs) originate from monocytes. The recruitment of monocytes to tumors requires chemokines and cytokines derived from tumor cells and stromal cells. One of the chemokines, [C-C motif] ligand 2 (CCL2), protects monocytes from apoptosis and induces a high level of autophagy in TAMs [90]. The transition from monocytes into macrophages requires colony-stimulating factor 1 (CSF1), which upregulates and activates ULK1 to trigger autophagy [91]. CSF2 also facilitates the differentiation of monocytes. Autophagy in monocytes maintains a high-level during differentiation, since JNK signaling is activated to release Beclin 1 and inhibits Atg5 cleavage. Autophagy inhibition restrains the differentiation process [92]. In addition, autophagy is involved in macrophage polarization. Downregulated autophagy promotes M1 polarization and upregulated autophagy promotes M2 polarization in macrophages [93]. M1 macrophages stimulate immune responses, whereas M2 macrophages play an immunosuppressive role, indicating that autophagy is a potential target to modulate macrophage polarization toward the M1 phenotype [94].

### 4.2. Autophagy in Regulating Immune Checkpoint Molecules

Immune checkpoint molecules, including PD-1 and CTLA-4, play crucial roles in tumor immune tolerance, which is the main reason for the poor clinical outcomes of immunotherapy. These immune checkpoint molecules regulate tumor immune tolerance through autophagy. For example, activation of PD-1 by PD-L1 promotes immunologic tolerance, suppresses effector T cells and maintains tumor Tregs, boosting tumor survival [95]. Hence, elucidating the regulation of immune checkpoints by autophagy is of great significance in immunotherapy.

As a T cell checkpoint molecule, PD-1 inhibits T cell proliferation and impedes the recognition of tumor cells once activated by PD-L1. Clark et al. found that tumor cells initiate autophagy in response to anti-PD1 or anti-PD-L1 antibody treatment. Engagement of PD-1 to PD-L1 induces autophagy in nearby T cells [96]. A study indicated that inhibition of autophagy by the Sigma1 inhibitor leads to degradation of PD-L1 and impaired PD-1/PD-L1 interaction, suggesting the Sigma1 inhibitor as a promising tool to block PD-1/PD-L1 [97]. Therefore, combining antibodies with autophagy inhibitors may be an attractive therapeutic strategy [98].

CTLA-4 was confirmed to be another immune checkpoint molecule that mediates tumor immune tolerance. Shukla et al. identified a subcluster of MAGE-A cancer-germline antigens that confer resistance to the CTLA-4 blockade. Autophagy activation combats this resistance by decreasing MAGE-A protein levels in human melanomas [99]. However, another study reported that mTORC1 inhibitor-induced autophagy restores CTLA-4 expression and corrects Treg cell function in systemic lupus erythematosus (SLE) [100]. CTLA4 engagement inhibits autophagy by activating PI3K/Akt/mTOR signaling pathway [101].

IDO promotes immunologic tolerance by suppressing responses of CTLs and maturation of DCs, magnifying tolerogenic APCs, and inducing Tregs generation. Autophagy can prevent inflammation-induced IDO synthesis by reducing inflammation [102]. IDO triggers the activation of general control nonderepressible 2 (GCN2) and inhibition of eukaryotic translation initiation factor 2α (eIF2α), which participate in inflammatory carcinogenesis [103]. Autophagy induced by IDO1-GCN2 plays a protective role against fatal inflammation disease [104]. Another mechanism of IDO-induced autophagy has been reported. The inhibition of tryptophan sufficiency signaling by IDO causes mTOR inactivation, thus triggering autophagy in an LC3-dependent way [103].

### 4.3. Autophagy in Immune Cytokines

Autophagy can increase or inhibit cytokine production and affect tumor progression. Autophagy and cytokines can regulate each other. Numerous cytokines have been demonstrated to govern or be governed by autophagy.

#### 4.3.1. Interleukins

IL-1 is a class of pro-inflammatory cytokines that promotes cancer progression by inhibiting COX-1, IkB, and JNK signaling pathways. Inhibition of IL-1 in tumor cells restrains tumor development [105]. IL-1β is negatively regulated by autophagy in most situations, whereas IL-1α is only negatively regulated by autophagy [106]. However, both IL-1α and IL-1β triggers autophagy, indicating that autophagy is a negative feedback mechanism in regulating IL-1 [107]. Autophagy activation in Atg5-deficient macrophages causes decreased IL-1β secretion, suppressing T cell activation and cytokine production [87]. Conversely, IL-1 level is elevated in autophagy-deficient macrophages to promote tumorigenesis through ROS-NF-κB signaling pathway [108]. IL-2 induces autophagy in an ATG5, HMGB1, and Beclin1-dependent way [109]. High-dose interleukin-2 (HDIL-2) inhibits the growth of metastatic liver tumors in vivo, accompanied by elevated levels of HMGB1, IFN-γ, IL-6, and IL-18 in serum. HMGB1 is translocated from the nucleus to the cytosol to trigger autophagy, and suppression of autophagy by CQ may enhance the proliferation and infiltration of immune cells in the liver and spleen [110]. IL-6 negatively regulates autophagy by activating STAT3 and inhibiting LC3-II and Beclin 1 expression in vitro [111]. However, IL-6 trans-signaling stimulates autophagy when IL-6 interacts with soluble IL-6R in vivo, which is mediated by AMPK and AKT activation [112]. In addition, IL-10 is also an autophagy suppressor by activating the JAK/STAT3 and PI3K/Akt/mTORC1 pathway [113]. As Th2 cytokines, IL-4, IL-13, and IL-10 can activate the PI3K/mTORC1 pathway to inhibit autophagy in most environments [114]. In turn, autophagy may exert dual effects on the production of IL-10, which needs further investigation [115].

#### 4.3.2. Interferons

Autophagy is needed in the synthesis of IFN-γ. IFN can be categorized into type I, II, and III. Type I IFN directly induces autophagy by activating JAK/STAT pathway [116]. IFN-α is a class of type I IFN, and the effect of IFN-α varies on different cell types [117]. As a type II IFN, IFN-γ induces autophagy in various types of immune cells and tumor cells, which is mediated by the acceleration of autophagosome formation and maturation [118]. In addition, IFN-γ can facilitate the MHCI expression and induce autophagy [119]. It has been reported that effector CD4+ T cells lacking Atg7 express low amounts of IFN-γ in the absence of autophagy [120].

#### 4.3.3. Transforming Growth Factor Beta (TGF-β)

TGF-β contributes to immune evasion by directly suppressing effector cells and indirectly facilitating the differentiation of Treg cells. TGF-β can also inhibit NK cells and IFN-γ to form immunosuppressive TME [121]. Inhibition of autophagy upregulates TGF-β expression by impairing its degradation [122]. In turn, TGF-β is responsible for autophagy induction in cancer cells [117]. For example, TGF-β induces autophagy in HCC and breast cancer cells by increasing the levels of ATGs, including Beclin1, Atg5, and Atg7. Autophagy promotes the expression of proapoptotic genes such as Bim and Bmf in the Bcl-2 family to mediate apoptosis, suggesting the correlations between autophagy and apoptosis [123,124].

#### 4.3.4. Tumor Necrosis Factor Alpha (TNF-α)

TNF-α is an apoptosis and necrosis inducer in multiple cells and can impair autophagy by decreasing lysosomal acidification [125]. Inhibition of autophagy by Bafilomycin A1 contributes to TNF-α-induced cell death by increasing oxidative stress and toxicity, indicating that autophagy is a negative regulator of TNF-α [126]. A mechanism study revealed that autophagy inhibits TNF-α expression through p38MAPK dephosphorylation and TRAF6 downregulation [127].

## 5. The Bidirectional Role of Autophagy in Immunotherapy

Whether autophagy plays a protective or destructive role in tumor cells or other cells depends on the degree of autophagy. Autophagy is a key player in regulating tumor immunity by affecting cells and cytokine release in tumor immunotherapy. Autophagy maintains this characteristic in TME. Although the direct connection between autophagy and immunotherapy has not been excessively explored, accumulating evidence indicates that autophagy may exert divergent effects on the tumor response to immunotherapy. The efficiency of immunotherapy may be enhanced or attenuated by autophagy, making autophagy a key player and a potential target for improving immunotherapy efficiency. In this section, we discuss the bidirectional role of autophagy in response to immunotherapy and demonstrate that modulation of autophagy can directly or indirectly enhance immunotherapy efficacy [81].

### 5.1. Autophagy Enhances the Effects of Immunotherapy

In immunotherapy, ferroptosis is one of the key mechanisms of tumor cell death mediated by CD8+ T cells [128]. A study demonstrated that knockout or knockdown of Atg5 and Atg7 prevents cancer cells from ferroptosis by decreasing intracellular ferrous iron levels and lipid peroxidation, indicating that autophagy may activate ferroptosis to enhance tumor immunotherapy [129,130]. Triple-negative breast cancer (TNBC) is usually characterized by autophagy defects, which inhibit T cell-mediated tumor killing in vitro and in vivo by blocking Tenascin-C degradation. Consequently, Tenascin-C inhibition sensitizes autophagy-defective TNBC cells to T cell killing and enhances the therapeutic effect of a single anti-PD1/PDL1 treatment. Based on these results, the researchers proposed a combination of Tenascin-C blockade and immune checkpoint inhibitors as a promising TNBC treatment [131].

As a supplement to immunotherapy, immunogenic cell death (ICD) is induced by many antitumor therapies to trigger antitumor immune responses. Autophagy is activated when ICD inducers are applied. In colon tumors, mitoxantrone or oxaliplatin treatment leads to autophagy in tumor cells, contributing to the infiltration of dendritic cells and T cells [132]. Mechanistically, T cell immunity is activated through antigen-presenting cells (APCs)-provided tumor antigens and DAMPs harbored in autophagosomes and released from dying cells [39]. Additionally, mitoxantrone-induced autophagy is also associated with the secretion of ATP in CT26 colon cancer cells. Extracellular ATP then recruits APCs and facilitates the synthesis of IL-1β to trigger an antitumor adaptive immune response [133]. Besides chemotherapy, autophagy may also contribute to the ICD to amplify the efficacy of radiotherapy. Ko et al. found that autophagy-induced ATP release from stressed or dying tumor cells triggers an anticancer immune response during radiotherapy [134]. The involvement of autophagy in targeted therapy has also been emphasized. As a tyrosine kinase inhibitor, sunitinib activates p62-dependent selective autophagy, thus inhibiting tumor PDL1 expression and facilitating anticancer immunity in metastatic breast cancer [135].

### 5.2. Autophagy Attenuates the Effects of Immunotherapy

Accumulating evidence indicate that hypoxia-induced autophagy is a mechanism related to immunotherapy failure that impairs CTLs-mediated tumor killing linked to STAT3 phosphorylation [136]. Additionally, hypoxia-induced autophagy eliminates NK cell-derived GrzB and weakens NK-mediated lysis in vitro and in vivo, indicating that autophagy may counteract immunotherapies that stimulate NK cells [57]. Tara et al. found that autophagy confers the resistance of cancer cells to immunotherapy. They discovered that deletion of Rb1cc1, an autophagy-related gene, sensitizes tumor cells to T cell killing, improving immune checkpoint blockade efficacy in mouse models [137]. Another study demonstrated the pleiotropic effects of autophagy in immunotherapy. It reported that knockout of ATG12 sensitizes cancer cells to CTLs, whereas knockout of ATG5 or ATG16L1 together with ATG12 results in resistance of cancer cells to CTLs [138].

Autophagy also participates in the indirect modulation of tumor immunity. When cancer cells undergo programmed cell death in response to various cell death inducers, dying cells release DAMPs into TME to activate immune response [53]. Inhibiting autophagy can facilitate this process, suggesting that a combination of autophagy inhibitors and cell death inducers can promote the anti-tumor effect of indirect immunotherapy, which has been verified in some animal models [139,140].

## 6. The Strategies of Targeting Autophagy to Facilitate Cancer Immunotherapy

Immunotherapy has demonstrated its great potential in cancer treatment and entered a new era after decades of development. Since autophagy plays a vital role in regulating tumor immune response, targeting autophagy appears to be a promising strategy to enhance immunotherapy efficacy and overcome immunotherapy resistance.

It has been reported that combining immunotherapy with radiotherapy or chemotherapy displays better efficacy than treatment alone. Radiotherapy or chemotherapy-induced autophagy contributes to augmenting mannose-6-phopsphate receptors (MPRs) in the tumor cell surface by autophagosome transportation, which increases the effectiveness of immunotherapy by making tumor cells more sensitive to CTLs [141,142]. Other research found that a semisynthetic vitamin E derivative, alpha-tocopheryloxyacetic acid (α-TEA), is an autophagy inducer that can aid in the presentation of tumor antigens to CD8+ T cells, thus improving anti-tumor immunity and immunotherapy efficacy [143]. Shen et al. designed a new probe, lactosylated N-Alkyl polyethylenimine-coated superparamagnetic iron oxide (SPIO) nanoparticles. They induced autophagy to promote DC maturation, indicating that SPIO nanoparticles may be a promising tool to enhance the vaccine functions of DCs [144]. Additionally, shikonin-induced autophagy can lead to DAMPs upregulation and activation of DCs [145]. Furthermore, the efficacy of DNA vaccines can be enhanced by synthesizing intracellular vaccine-encoded tumor antigens [146]. SQSTM1/p62 is a new cancer antigen connected to autophagy. The anti-tumor and anti-metastatic effects of p62-encoding DNA vaccines have been detected, and may be a potential method for tumor immunotherapy [147].

**Figure 3:**
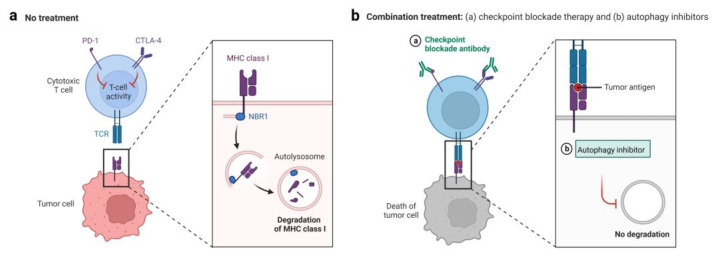
Autophagy blockage sensitizes cancer cells to immunotherapy. (**a**) In tumor cells, surface MHC-I is degraded by the autophagy-lysosome traffic, facilitating malignant cells to evade immunologic surveillance. (**b**) Blockage of autophagosome or lysosome intercepts MHC-I degradation, enhancing efficacy of checkpoint blockage inhibitors.

In melanoma and colorectal cancer, vacuolar protein sorting 34 (VPS34) inhibition by SB02024 or SAR405 upregulates the level of CCL5, CXCL10, and IFNγ in the TME, resulting in increased tumor infiltration of NK cells and T cells. Combining treatment of SB02024 or SAR405 with either anti-PD-1 or anti-PD-L1 prolonged survival of mice nearly 9 days in ananti-PD-1/PD-L1 treatment alone, indicating that the therapeutic efficacy of anti-PD-L1/PD-1 is improved by VPS34 inhibitor, which terminates autophagosome formation [148] (Table 1). Lawson et al. also reported that a VPS34 inhibitor, autophinib, sensitizes various tumor cell lines to TNFα [138] (Table 1). Sharma et al. found that anti-PD-1 Ab and HCQ decreased the tumor volume and increased the survival of mice compared with anti-PD-1 Ab alone. They proposed that targeting palmitoyl-protein thioesterase 1 (PPT1) by hydroxychloroquine (HCQ) or genetic Ppt1 inhibition can enhance the anti-PD-1 Ab efficacy in melanoma, which may be mediated by lysosome dysfunction [149] (Table 1). Other research demonstrated that autophagy inhibition by CQ improves antigen presentation and anti-tumor T cell responses by restoring surface levels of MHC-I [55] (Figure 3, Table 1). Yu et al. proposed an osteosarcoma treatment strategy by combination of the autophagy inhibitor 3-MA and photodynamic therapy (PDT), which induces an immunological response by downregulating PD-L1 expression, leading to tumor growth block in vitro and in vivo [150]. HDIL-2 is often applied for melanoma and renal cell carcinoma treatment, but this treatment easily causes side effects. Autophagy inhibitor CQ strengthens the effectiveness of HDIL-2 immunotherapy and reduces the toxicity in metastatic liver cancer [110] (Table 1). Similarly, the efficacy of HDIL-2 immunotherapy in treating renal cell carcinoma is improved by CQ by enhancing the functions of DCs, T cells, and NK cells, limiting ATP production, and increasing apoptosis [151] (Table 1). Other research discovered that the autophagy inhibitor 3-MA significantly promotes IL-24-induced apoptosis in oral squamous cell carcinomas (OSCC), suggesting that combining 3-MA and IL-24 may be a potential method in immunotherapy. Inhibition of autophagy can recover deficient ICD-based cancer immunotherapy. However, autophagy inhibition resulted in a loss of ATP and hampered the antitumor immune response. Hence, Li et al. proposed a novel coping strategy by introducing ATP as a remote loading gradient of the liposome to encapsulate HCQ (LipHCQa), which displayed a potent anti-colon cancer effect without restraining the immune response [152] (Table 1). The combination of HCQ and radiation therapy can enhance ICD in patients with glioblastoma multiforme [153]. Meanwhile, in combination with other therapies, autophagy inhibition can improve immune activity. For example, the results of a clinical trial indicated that gemcitabine and nab-paclitaxel chemotherapy in combination with HCQ treatment increased the infiltration of immune cells in patients with resectable pancreatic adenocarcinoma [154]. However, the lack of specificity in autophagy inhibitors needs to be addressed. CQ and HCQ may exert suppressive effects on immune responses to IDO1 and PD-L1 [155].

In addition to targeting autophagy itself, targeting the signaling that regulates autophagy has the potential to improve immunotherapy. For example, Wu et al. found that combination of the PD-1 blockade and endostar remarkably inhibited Lewis lung carcinoma (LLC) growth by activating PI3K/AKT/mTOR-mediated autophagy [156]. Conversely, inhibition of mTOR triggers autophagy, which mediates the resistance of tumor cells to T cell killing [137]. These facts suggest that mTOR is a promising autophagy-related target that may aid in immune system activation. Moreover, RAS, RAF, AMPK, and STAT3 are regarded as autophagy modulators to affect immunotherapy efficacy [4]. However, the highly context-dependent roles of these targets must be taken into consideration.

As a type of immunotherapy, CAR T cell therapy is clinically effective in treating hematologic cancers but is ineffective in treating solid tumors [157]. This may result from the TME of solid tumors, which creates a barrier to infiltration and the function of CAR T cells. Autophagy regulation is a recently proposed strategy to improve the efficacy of CAR T cell therapy since autophagy is closely related to TME and chemokine synthesis [158]. On the one hand, autophagy inhibition has been confirmed to promote CAR T cell tumor trafficking. CAR T cells eliminate tumor cells by recognizing cell surface antigens which can be degraded by autophagy [159]. Hence, inhibiting autophagy in tumors may enhance CAR T therapy efficacy by increasing the expression of antigens. On the other hand, the survival and normal function of T cells requires a high level of autophagy to deal with metabolic stress in TME [160]; therefore, strengthened autophagy in CAR T cells before transfusion to patients may improve the therapeutic outcome.

## 7. Perspectives

Currently, immunotherapies have produced favorable clinical outcomes. However, many patients do not respond, or develop resistance, to immunotherapy. Autophagy is a key regulator of immune responses in TME. The bidirectional roles of autophagy in cancer progression have been well established, which is context-dependent and tumor type-dependent. Autophagy in cancer cells or immune cells may improve or attenuate the effects of immunotherapy. Due to the insufficient understanding of the complexity of autophagy, the development of autophagy-base monotherapy or combined therapy is challenging. Hence, whether autophagy exerts pro-survival or pro-death effects, and how to enhance or inhibit it at different stages and in different cells, warrants further study. Some autophagy activators and inhibitors are being tested in preclinical and clinical studies. The combinations of autophagy modulators and various therapies have been confirmed to be effective, but only a few examples are immunotherapies. Since targeting autophagy appears to be an option in combinatorial therapy from which patients my benefit, further efforts should be taken to explore how to enhance tumor immunogenicity, improve T cell function, and weaken immunosuppressive TME by targeting autophagy.

## Figures and Tables

**Table 1:** Autophagy inhibitors enhance the efficacy of immunotherapy.

Autophagy Inhibitor	Targets	Immunotherapy	Tumor Types	Refs.
SB02024	Vps34	Anti-PD-L1 and Anti-PD-1	Melanoma, CRC	[148]
SAR405	Vps34	Anti-PD-L1 and Anti-PD-1	Melanoma, CRC	[148]
Autophinib	Vps34	TNFα	Various cancers	[138]
Hydroxychloroquine	Lysosomes, PPT1	anti-PD-1	Melanoma	[149]
LipHCQa	Lysosomes	Shikonin-induced ICD	Colon cancer	[152]
Chloroquine	Lysosomes	anti-PD1/CTLA4 HDIL-2	Pancreatic cancer, metastatic liver cancer, renal cell carcinoma	[55,110,151]

## Data Availability

Not applicable.
